# The Amigo™ Remote Catheter System: From Concept to Bedside

**DOI:** 10.19102/icrm.2017.080806

**Published:** 2017-08-15

**Authors:** Zohaib A. Shaikh, Michael F. Eilenberg, Todd J. Cohen

**Affiliations:** ^1^Department of Medicine, NYU Winthrop Hospital, Mineola, NY; ^2^Vicarious Surgical, Inc., Cambridge, MA

**Keywords:** Amigo remote catheter system, clinical experiences, clinical trials, radiofrequency ablation, robotic surgery

## Abstract

Radiation exposure is a serious concern during fluoroscopic procedures, including electrophysiology (EP) studies and radiofrequency catheter ablation of arrhythmias. Operators typically don lead aprons to protect themselves from radiation, but wearing lead can result in greater fatigue and orthopedic injury during long procedures. To address this problem, two robotic catheter systems (RCS) have previously been introduced on the market, the Niobe^®^ (Stereotaxis Inc., St. Louis, MO, USA) and Sensei^®^ X (Hansen Medical, Inc., Mountain View, CA, USA) systems. However, the widespread adoption of these systems has been limited by both cost and ease of use. In contrast, the Amigo™ RCS (Catheter Precision, Inc., Mount Olive, NJ, USA) was developed to provide a simple, lower profile, and less expensive remote catheter manipulation solution. Approved by the United States Food and Drug Administration (FDA), this technology allows for operators to remotely manipulate electrophysiology (EP) catheters from outside the fluoroscopy field. Notably, the Amigo™ RCS (Catheter Precision, Inc., Mount Olive, NJ, USA) first underwent an early study in dogs in 2008 to demonstrate its safety and efficacy in an animal model. After a clinical trial evaluating its safety and mapping capabilities in humans was completed in 2010, the Amigo™ RCS (Catheter Precision, Inc., Mount Olive, NJ, USA) underwent several scientific studies to examine its ability to assist in the mapping and ablation of various arrhythmias in comparison with the conventional manual approach. The Amigo™ RCS (Catheter Precision, Inc., Mount Olive, NJ, USA) achieved mapping and ablation success rates that were similar to those achieved with manual catheter manipulation, and no complications due to its use were observed. It was approved by the FDA for use in diagnostic EP studies of the right atrium and ventricle in 2012, with this indication later expanded in 2014 to include radiofrequency ablations. The device is currently compatible with the Blazer™ (Boston Scientific, Natick, MA, USA) and EZ STEER™ (Biosense Webster, Inc., Diamond Bar, CA, USA) catheter handles. Here, we present a clinical report in which the Amigo™ RCS (Catheter Precision, Inc., Mount Olive, NJ, USA) was employed to map and ablate symptomatic supraventricular tachycardia. Dr. Cohen’s clinical experience with this robotic system is also reviewed.

## Introduction

Contemporary electrophysiology (EP) studies traditionally evaluate the conduction and refractoriness of the heart, as well as its propensity to demonstrate tachyarrhythmias, in a minimally invasive fashion through the use of percutaneous catheters that are placed and positioned fluoroscopically in the heart. The mapping and ablation components of complex arrhythmias lengthen the total procedural time significantly and add to the operator’s cumulative radiation exposure.^1–6^ Operators typically wear lead, or lead-like, aprons in order to reduce their exposure to radiation. However, the weight of such shielding can lead to fatigue and orthopedic injury, including disc herniation and the onset of back or neck pain.^6–8^

### The unmet need

In light of this, there was an unmet need noted for a simple, inexpensive method of remotely manipulating ablation catheters in order to minimize the operator’s exposure to radiation and the degree of wear and tear of the operator’s neck and spine.

### Remote catheter systems

Two remote catheter systems have previously been developed and approved by the United States Food and Drug Administration (FDA) to address the issue of operator radiation exposure. The Niobe^®^ magnetic navigation system (Sterotaxis, Inc., St. Louis, MO, USA) utilizes two computer-controlled permanent magnets to create a magnetic field in the patient’s chest.^9,10^ This system requires the use of flexible catheters that incorporate a permanent magnet inside the tip, to allow for the manipulation of their movement through the adjustment of the magnetic field’s orientation.^9–11^ During use, the catheter is advanced and withdrawn using a motor-driven unit (Cardiodrive^®^; Sterotaxis, Inc., St. Louis, MO, USA) and is maneuvered by the operator, who is in the control room, through a video workstation (Navigant™; Sterotaxis, Inc., St. Louis, MO, USA).^9,10^ The Niobe^®^ system (Sterotaxis, Inc., St. Louis, MO, USA) is exceptionally accurate in achieving catheter positioning and is unlikely to cause cardiac perforation^11^ since it utilizes a soft catheter that is pulled towards the endocardium by an external magnetic field, rather than a mechanically driven rigid catheter. However, this system is very expensive, and a physically large EP laboratory is required to accommodate its sizeable platform.

The Sensei^®^ Robotic System (Hansen Medical, Mountain View, CA, USA) uses a robotic steerable sheath system (Artisan^®^ Extend Control Catheter; Hansen Medical, Mountain View, CA, USA) to manipulate catheters within the heart.^12^ The system consists of a large workstation with an instinctive motion controller that registers the movements of the operator’s hand. Using a master–slave control system, these movements are translated into three-dimensional (3D) motions of the catheter by the remote catheter manipulator and steerable sheath. An early ex vivo study demonstrated that the Sensei^®^ X system (Hansen Medical, Mountain View, CA, USA) was able to successfully navigate and complete precise movements more quickly than could be done through using manual catheter manipulation.^13^ A later human trial confirmed that the Sensei^®^ X system (Hansen Medical, Mountain View, CA, USA) is safe and feasible to be used for the mapping and ablation of atrial fibrillation (AF) and atrial flutter (AFL).^12^ Unlike the Niobe^®^ magnetic navigation system (Sterotaxis, Inc., St. Louis, MO, USA), the Sensei^®^ X Robotic System (Hansen Medical, Mountain View, CA, USA) can passively accommodate any mapping or ablation catheter within its steerable sheath. However, the system requires the percutaneous insertion of a large, specifically designed 14F steerable sheath (Artisan^®^ Catheter; Hansen Medical, Mountain View, CA, USA) in order to contain the shaft of a much smaller (8F) ablation catheter. The invasiveness and expense of this sheath and system are potential barriers to widespread adoption of this technology within the EP community. Thus, these two systems only partially represent solutions to the previously stated unmet need.

### Concept

**Figure 1:**
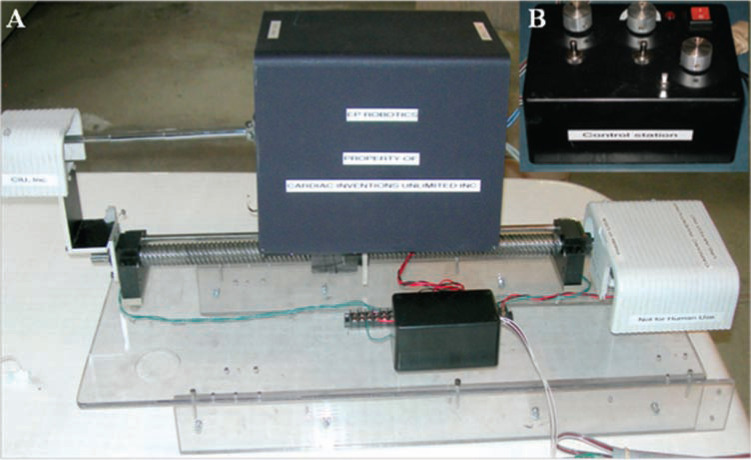
Early prototype of the RCS. **A:** The remote catheter system containing motors, capable of manipulating the installed catheter in three dimensions. **B:** A control station, which uses switches and dials to steer the aforementioned motors.

Owing to the existing concerns regarding radiation exposure to operators, along with the limitations of existing solutions, Dr. Todd Cohen, an author, attempted to create a more accessible solution, with respect to cost and size. In 2005, he and Dr. Michael Eilenberg (the latter an engineering student at Rensselaer Polytechnic Institute in Troy, New York, at the time) conceived and developed a system that is capable of remotely manipulating commercially available catheters through an external robotic device. This system utilizes the catheters’ internal steering mechanisms, in contrast to the external robotic steerable sheath system used in the Sensei^®^ X (Hansen Medical, Mountain View, CA, USA).^14^ Dr. Eilenberg and fellow students built an early prototype based on the developed designs **(Figure 1)**, which demonstrated proof-of-concept. In 2006, Catheter Robotics, Inc. was formed around this novel robotic system. The company later changed its name to Catheter Precision, Inc. in 2016.

**Figure 2:**
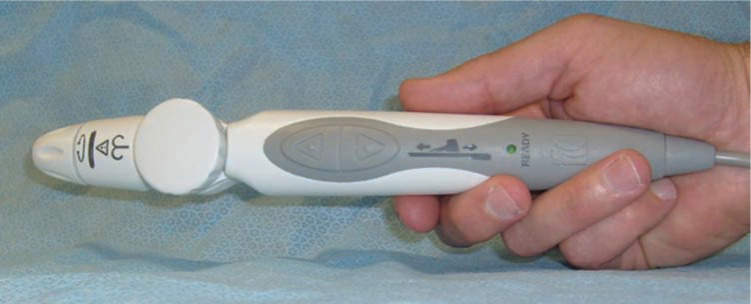
The Amigo™ RCS (Catheter Precision, Inc., Mount Olive, NJ, USA) remote controller. The design resembles a traditional catheter handle and allows for the user to remotely advance, withdraw, rotate and deflect the tip of an attached standard mapping or ablation catheter as desired.

**Figure 3:**
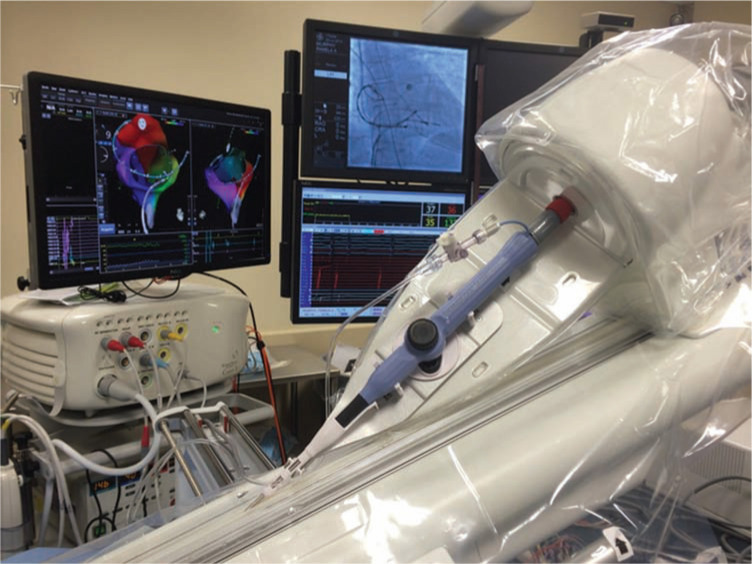
The Amigo™ RCS (Catheter Precision, Inc., Mount Olive, NJ, USA) robotic catheter manipulator. During use, a traditional catheter is installed into the manipulator, which advances, withdraws, rotates, and deflects the tip of the catheter as needed according to the operator’s input, which is transmitted via the controls on the handle. The manipulator is draped in a sterile fashion and the system is used in conjunction with the EnSite™ NavX™ electroanatomic mapping system (Abbott Laboratories, Chicago, IL, USA).

Following its formation, Catheter Robotics, Inc. built a new prototype, which maintained functionality similar to that of the earlier version, in order to adapt the concept for clinical applications and named it the Amigo™ Remote Catheter System (RCS). The redesigned device was comprised of a handheld remote controller that resembles a traditional catheter handle **(Figure 2)** and a sleek robotic catheter manipulator capable of holding different manufacturers’ mapping and ablation catheters and operating in a sterile fashion. The bridge that holds the catheter is attached to the operating table **(Figure 3)**. In contrast to the Niobe^®^ (Sterotaxis, Inc., St. Louis, MO, USA) and the Sensei^®^ X (Hansen Medical, Mountain View, CA, USA), the Amigo™ (Catheter Precision, Inc., Mount Olive, NJ, USA) does not require a separate workstation or proprietary catheters or sheaths to function. It is therefore less expensive to implement and can easily be integrated into an existing EP laboratory. A unique aspect of the Amigo™ RCS (Catheter Precision, Inc., Mount Olive, NJ, USA) is its manual override feature, which allows for the installed catheter handle to quickly be removed from the system for manual operation, and then re-attached to the system without compromising catheter sterility or positioning, providing the operator with maximum flexibility during complex cases.^15^

### Trials

***Feasibility in animals.*** In 2008, Knight et al.^16^ conducted a study aimed at assessing the safety and efficacy of the Amigo™ RCS (Catheter Precision, Inc., Mount Olive, NJ, USA) in a canine model, in comparison with the manual operation of a catheter. The study tested the system’s ability to allow its operator to position the attached EP catheter in five different sites in the heart and investigated whether there was any potential for cardiac perforation or injury. Pacing thresholds were measured to assess endocardial contact. Ultimately, the operator was able to successfully manipulate the catheter into all target positions, with no significant difference in pacing thresholds, electrogram amplitude, or interval measurements between robotic and manual catheter operation, indicating similar and adequate contact with the cardiac tissue. No adverse events or cardiac injury were observed during the study, even with the use of excessive force, due to the preservation of catheter buckling.

***Mapping.*** The Amigo™ RCS (Catheter Precision, Inc., Mount Olive, NJ, USA) received investigational device exemption (IDE) approval and underwent its first clinical trial (NCT01139814) beginning in 2010. The study, conducted by Khan et al.,^17^ evaluated the safety and performance of the Amigo™ (Catheter Precision, Inc., Mount Olive, NJ, USA) in mapping the right side of the heart. Eight sites were mapped in 181 patients, with a success rate of 96% according to fluoroscopic and electrical criteria, with no complications resulting from the use of the Amigo™ RCS (Catheter Precision, Inc., Mount Olive, NJ, USA). These results also indicated only a short learning curve was needed for the handling of the device. The first procedure had the longest average manipulation time, but this length of time was observed to have significantly decreased by the third procedure in the study. A significant decrease in fluoroscopy time was also observed by the eighth case. While 92% of physicians agreed that the Amigo™ RCS (Catheter Precision, Inc., Mount Olive, NJ, USA) was easy to operate, and 77% reported ease of catheter control after the completion of their first case, the prevalence of these sentiments grew to 97% and 96%, respectively, by the end of the study.

***Ablation.*** A study by Datino et al.^18^ in 2014 aimed to evaluate the safety and feasibility of using the Amigo™ RCS (Catheter Precision, Inc., Mount Olive, NJ, USA) versus manual catheter manipulation to ablate a variety of arrhythmias encountered in their EP laboratory. Two operators used the Amigo™ (Catheter Precision, Inc., Mount Olive, NJ, USA) to complete ablations in 50 consecutive patients at a single center. The researchers matched and compared the results of these cases to those of a control group of 50 patients who underwent the same ablation procedure completed by the same operators during the same time period but with using manual ablation. Both techniques were used to ablate cases involving atrioventricular (AV) node reentrant tachycardia, common AFL, accessory pathways, AF, ventricular tachycardia, and atypical AFL/atrial tachycardia. There were no significant differences noted between the case success rate achieved using the Amigo™ RCS (Catheter Precision, Inc., Mount Olive, NJ, USA) versus those treated with manual ablation. The ablation procedure succeeded in 96% of patients using only the Amigo™ RCS (Catheter Precision, Inc., Mount Olive, NJ, USA). In one case, the operator decided to switch to cryotherapy, which required manual operation, as cryoablation catheters are not yet compatible with the Amigo™ RCS (Catheter Precision, Inc., Mount Olive, NJ, USA). In another case, the physician decided to continue the ablation manually, due to difficulties noted in crossing the patient’s aortic valve to ablate a left-sided ventricular tachycardia. However, no complications due to the use of the Amigo™ RCS (Catheter Precision, Inc., Mount Olive, NJ, USA) were observed. Procedure duration and radiofrequency delivery time also did not significantly differ between the robotic and manual ablation procedures. The use of the Amigo™ RCS (Catheter Precision, Inc., Mount Olive, NJ, USA) was associated with an 86% reduction in average operator radiation exposure time.

A multicenter study also conducted in 2014 assessed the effectiveness of cavotricuspid isthmus (CTI) ablation using the Amigo™ RCS (Catheter Precision, Inc., Mount Olive, NJ, USA) in 60 patients with typical AFL.^19^ Operators achieved stable, bidirectional CTI block in 59 of the 60 patients, for a success rate of 98%. In one patient, the physician switched to manual operation of the catheter when block was not achieved after 43 minutes of radiofrequency application. The use of a non-fluoroscopic mapping system (EnSite™; Abbot Laboratories, Chicago, IL, USA) in tandem with the RCS in one center reduced the fluoroscopy time significantly but increased the total duration of the procedure.

In a single-center analysis, there were no significant differences in radiofrequency application time, fluoroscopy time, or total procedure duration between the first 10 cases and the final 24 cases.

A dual-center study in 2014 by Wutzler et al.^20^ examined the feasibility of using the Amigo™ RCS (Catheter Precision, Inc., Mount Olive, NJ, USA) for left atrial mapping and pulmonary vein isolation (PVI) in patients with symptomatic paroxysmal AF versus using a traditional manual approach. PVI was successful in all patients, regardless of whether the RCS or manual approach was used. There were no significant differences in total fluoroscopy time, total energy delivery, or length of procedure. However, the operator’s fluoroscopy exposure was significantly reduced in the RCS group. This study encountered no procedural complications. Furthermore, an analysis of the first 20 patients found that the latter 10 cases (cases 11 to 20) included a significantly reduced procedure duration, but did not have any significant differences in terms of total fluoroscopy time or operator fluoroscopy exposure in comparison with the first 10 cases, reaffirming that use of the Amigo™ RCS (Catheter Precision, Inc., Mount Olive, NJ, USA) includes a short learning curve.^21^

### Clinical uses

***Regulatory approval.*** Based on clinical data from the IDE clinical trial,^16^ 510(k) approval was granted by the FDA in June 2012.^22^ This allowed for the Amigo™ RCS (Catheter Precision, Inc., Mount Olive, NJ, USA) to be marketed for providing assistance in the positioning and manipulation of diagnostic catheters in the right atrium and right ventricle during EP studies. This notification limited the use of the Amigo™ RCS (Catheter Precision, Inc., Mount Olive, NJ, USA) to diagnostic studies and mapping done using the Blazer™ Dx-20 catheter (Boston Scientific, Natick, MA, USA), as adequate safety and effectiveness data had not yet been collected for the RCS’s use with any other commercially available catheters. The 510(k) clearance was expanded in November 2012 to include pairing with the EZ STEER™ diagnostic catheters (Biosense Webster, Inc., Diamond Bar, CA, USA).^23^ EP laboratories in the US have been able to use the Amigo™ RCS (Catheter Precision, Inc., Mount Olive, NJ, USA) in right-sided EP studies and mapping since 2012.

In 2014, the FDA approved a de novo submission by Catheter Robotics, Inc. requesting the ability to expand indications for the use of the Amigo™ RCS (Catheter Precision, Inc., Mount Olive, NJ, USA) in right-sided radiofrequency catheter ablations.^24,25^ Based on clinical studies, the Amigo™ RCS (Catheter Precision, Inc., Mount Olive, NJ, USA) demonstrated high acute and chronic ablation success rates in comparison with those of the traditional manual approach. While fluoroscopy time and procedure duration were similar for both approaches, the use of the Amigo™ RCS (Catheter Precision, Inc., Mount Olive, NJ, USA) resulted in a significant reduction in radiation exposure to the operator. The system was thus deemed to yield the same benefits as manual ablation, while also providing the additional benefit of reducing the operator’s exposure to radiation.

Biosense Webster, Inc. also received approval in 2014 from the FDA to market the ThermoCool^®^ SmartTouch™ catheter, which introduced the ability to sense and record contact force between the myocardium and the catheter’s tip in real time.^26^ This feature was integrated into the CARTO^®^ 3 nonfluoroscopic mapping system (Biosense Webster, Inc., Diamond Bar, CA, USA). Using the Smart-Touch™ catheter (Biosense Webster, Inc., Diamond Bar, CA, USA) with the CARTO^®^ 3 system (Biosense Webster, Inc., Diamond Bar, CA, USA) allows operators to monitor the force exerted by a catheter remotely controlled by the Amigo™ RCS (Catheter Precision, Inc., Mount Olive, NJ, USA) on the myocardium. Previously contact force could only be estimated via fluoroscopic images and the magnitude of sensed electrograms.^27^

## Case presentation

**Figure 4:**
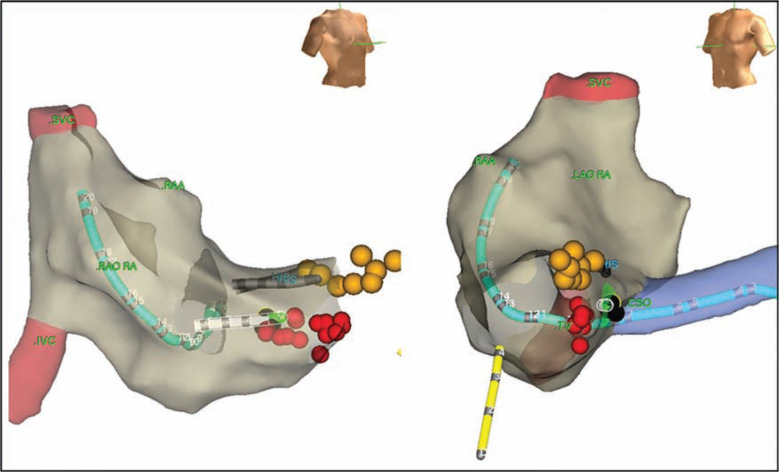
A 3D map of the right atrium (tan), along with the superior and inferior vena cavae (light red). The image to the left displays the right anterior oblique projection, while the image to the right displays the left anterior oblique projection. During the procedure, a duo-decapolar catheter was positioned in the coronary sinus, and the His-bundle was recorded at the sites indicated in yellow. Initial unsuccessful ablations were performed at the sites of the red markers. The image displays the short distance between the His-bundle recording and the coronary sinus ostium in this patient. Ablation performed at the more septal site, as indicated by the black markers, resulted in the termination of the tachycardia. These images were captured using the EnSite™ NavX™ Navigation and Visualization Technology (Abbott Laboratories, Chicago, IL, USA).

**Figure 5:**
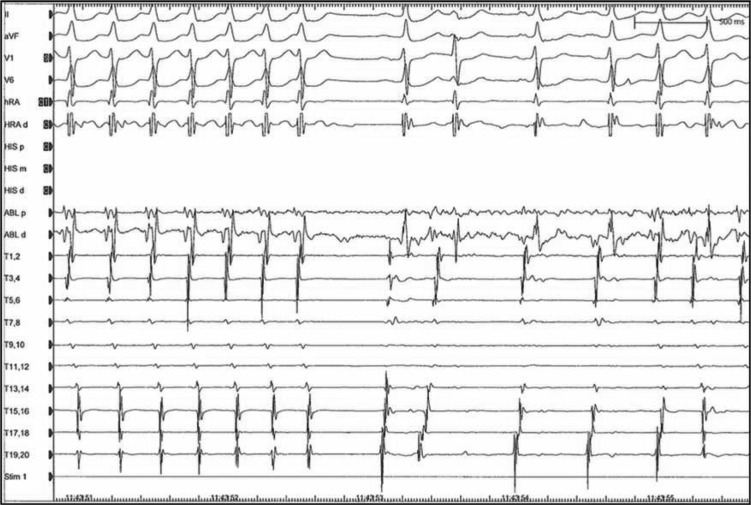
Surface and intracardiac electrograms from the EP study, showing the ablation of the supraventricular tachycardia, with termination of the tachycardia. ABL d shows the distal electrograms recorded during the delivery of radiofrequency energy. The earliest atrial electrograms in the distal ablation recording (ABL d) precede any other atrial electrograms recorded.

A 62-year-old man with a history of dramatic supraventricular tachycardia was referred for an EP study, which demonstrated AV node reentrant tachycardia, and subsequently underwent radiofrequency ablation with robotic assist. **Figure 4** shows a non-fluoroscopic image (EnSite™ NavX™ Navigation and Visualization Technology; Abbott Laboratories, Chicago, IL, USA) of the right atrium (tan) with the superior and inferior vena cavae, indicated in light red. A duo-decapolar catheter (7F Livewire™ Duo-Decapolar Electrophysiology Catheter; Abbott Laboratories, Chicago, IL, USA) was positioned in the coronary sinus, and the His-bundle was recorded at sites indicated by the yellow markers. The initial unsuccessful ablation approach included electroanatomic mapping in normal sinus rhythm in a posteroseptal location with a small atrial electrogram and approximately a four-fold larger ventricular electrogram. A slow pathway potential was not able to be recorded, and radiofrequency applications at the sites of the red markers failed to initiate a junctional rhythm **(Figure 4)**. The distance between the His-bundle recording (in yellow) and the coronary sinus ostium was particularly small in this patient, indicating a potential increased risk of damage to the patient’s fast pathway during a slow pathway ablation. Ultimately, the successful approach employed the Amigo™ RCS (Catheter Precision, Inc., Mount Olive, NJ, USA), which helped to stabilize a 7F, 4 mm extended distal Blazer™ II Temperature Ablation catheter tip (Boston Scientific, Natick, MA, USA) and precisely map the earliest retrograde atrial electrogram recording during tachycardia (indicated via the black markers in **Figure 4)**. Radiofrequency catheter ablation performed at that more septal location (black markers) resulted in the prompt termination of tachycardia **(Figure 5)** with a junctional rhythm, and no additional tachycardia was induced.

### NYU Winthrop Hospital experience

The Amigo™ RCS (Catheter Precision, Inc., Mount Olive, NJ, USA) has been used at NYU Winthrop Hospital for various cases by Dr. Cohen. The system is supplemented with commercially available electroanatomic mapping software to provide accurate, real-time data to non-fluoroscopically facilitate mapping and ablation. EP study reports pertaining to cases involving the Amigo™ RCS (Catheter Precision, Inc., Mount Olive, NJ, USA) were retrospectively analyzed. This analysis was submitted to the NYU Winthrop Hospital institutional review board and was approved as an exempted study. All procedures were performed in accordance with the principles of the Declaration of Helsinki.

A total of 45 right-sided mapping procedures or radiofrequency ablations were performed on 43 patients using the Amigo™ RCS (Catheter Precision, Inc., Mount Olive, NJ, USA). Of the 43 patients who underwent procedures, 23 (53.5%) were female. These patients had an average age of 53.4±19.4 years, while the male patients had an average age of 60.6±17.6 years. The types of ablation procedures performed, along with the average fluoroscopy time per procedure, are displayed in **Table 1**. The majority of cases involved the ablation of AFL (51.1%), and only four cases utilized the Amigo™ RCS (Catheter Precision, Inc., Mount Olive, NJ, USA) for mapping without a following ablation. Two patients underwent two consecutive, discrete ablations during a single case, one with ablations of AFL and the slow AV nodal pathway, and the other with ablations of AFL and a right posteroseptal bypass tract.

All 43 cases had successful outcomes, with no complications reported. The mean fluoroscopy time for all Amigo™ RCS (Catheter Precision, Inc., Mount Olive, NJ, USA) cases was 8.2±6.3 min. Two of the 43 cases (4.7%) utilized the manual override feature of the Amigo™ RCS (Catheter Precision, Inc., Mount Olive, NJ, USA). The EnSite™ NavX™ (Abbott Laboratories, Chicago, IL, USA) system was used in 27 cases (62.8%), while the CARTO^®^ 3 system (Biosense Webster, Inc., Diamond Bar, CA, USA) was used in six cases (14%). All six of the cases completed with the CARTO^®^ 3 system (Biosense Webster, Inc., Diamond Bar, CA, USA) also used the ThermoCool^®^ SmartTouch™ catheter (Biosense Webster, Inc., Diamond Bar, CA, USA), which enabled contact force sensing to be used. The electroanatomic mapping software used was not specified in 10 study reports (23.2%).

Additionally, the NYU Winthrop Hospital team has previously published two cases completed by Dr. Cohen, which have highlighted the benefits of contact force sensing and the manual override feature in the use of the Amigo™ RCS (Catheter Precision, Inc., Mount Olive, NJ, USA). One case involved the mapping and ablation of sustained clockwise AFL with the Amigo™ RCS (Catheter Precision, Inc., Mount Olive, NJ, USA) and the ThermoCool^®^ SmartTouch™ contact force sensing ablation catheter (Biosense Webster, Inc., Diamond Bar, CA, USA). Real-time contact force measurements provided valuable feedback in this case and were helpful during the procedure. The other report described a case with a difficult right ventricular outflow tract ventricular tachycardia ablation also performed using the Amigo™ RCS (Catheter Precision, Inc., Mount Olive, NJ, USA) and the ThermoCool^®^ SmartTouch™ catheter (Biosense Webster, Inc., Diamond Bar, CA, USA).^15^ High forces were recorded consistently while using the Amigo™ RCS (Catheter Precision, Inc., Mount Olive, NJ, USA) with this patient, and the manual override feature allowed for the safe and successful completion of this procedure.

## Conclusions

This paper reviewed the development of the Amigo™ RCS (Catheter Precision, Inc., Mount Olive, NJ, USA) from concept to bedside. Dr. Cohen has had the unique opportunity of conceiving this robotic system, helping to build the first prototype, helping to form the first company, being involved in the first animal study, and using the device clinically to perform right-sided ablation cases of supraventricular and ventricular tachyarrhythmias. The Amigo™ RCS (Catheter Precision, Inc., Mount Olive, NJ, USA) enables electrophysiologists to operate with less radiation exposure than manual procedures, without compromising clinical efficacy. In addition, the application of contact force sensing has added valuable feedback during remote mapping and ablation procedures, and the manual override feature is unique to this system. The low cost and small profile of the Amigo™ RCS (Catheter Precision, Inc., Mount Olive, NJ, USA) may facilitate its adoption within the EP community. Future studies should address the utility of this system in left-sided procedures, including the ablation of AF, left atrial tachycardia, left AFL, and left ventricular tachycardia.

## Acknowledgments

The authors would like to thank Matthew Oakley and Greg Russell, MBA, for assisting Dr. Eilenberg in building the first prototype of the remote catheter system in 2005.

## Figures and Tables

**Table 1:** Types of Ablations Performed with the Amigot Remote Catheter System

Type of Ablation	Number (%)	Mean Fluoroscopy Time (min)
AFL	24 (51.1)	8.4 ± 5.5
Atrial tachycardia	3 (6.7)	6.8 ± 3.1
VT (RVOT)	6(13.3)	13.8 ± 9.8
AVNRT	7(15.6)	4.3 ±2.7
AVRT	1 (2.2)	22*
N/A (mapping only)	4 (8.9)	4.0 ±2.8
Total	45 (100)	8.2 ±6.3

AFL: atrial flutter; AVNRT: atrioventricular nodal reentrant tachycardia; AVRT: atrioventricular reentrant tachycardia; N/A: not applicable; RVOT: right ventricular outflow tract; VT: ventricular tachycardia.

*Only one AVRT ablation was performed with the Amigo™ RCS (Catheter Precision, Inc., Mount Olive, NJ, USA); these data represent the total fluoroscopy time for one case in which both an AFL ablation and an AVRT ablation were completed.
